# Predictive Physiological Anticipation Preceding Seemingly Unpredictable Stimuli: A Meta-Analysis

**DOI:** 10.3389/fpsyg.2012.00390

**Published:** 2012-10-17

**Authors:** Julia Mossbridge, Patrizio Tressoldi, Jessica Utts

**Affiliations:** ^1^Department of Psychology, Northwestern UniversityEvanston, IL, USA; ^2^Dipartimento di Psicologia Generale, Università di PadovaPadova, Italy; ^3^Department of Statistics, University of CaliforniaIrvine, CA, USA

**Keywords:** pre-stimulus activity, anticipatory physiology, temporal processing, psychophysiology, presentiment, predictive processing

## Abstract

This meta-analysis of 26 reports published between 1978 and 2010 tests an unusual hypothesis: for stimuli of two or more types that are presented in an order designed to be unpredictable and that produce different post-stimulus physiological activity, the direction of pre-stimulus physiological activity reflects the direction of post-stimulus physiological activity, resulting in an unexplained anticipatory effect. The reports we examined used one of two paradigms: (1) randomly ordered presentations of arousing vs. neutral stimuli, or (2) guessing tasks with feedback (correct vs. incorrect). Dependent variables included: electrodermal activity, heart rate, blood volume, pupil dilation, electroencephalographic activity, and blood oxygenation level dependent (BOLD) activity. To avoid including data hand-picked from multiple different analyses, no *post hoc* experiments were considered. The results reveal a significant overall effect with a small effect size [fixed effect: overall ES = 0.21, 95% CI = 0.15–0.27, *z* = 6.9, *p* < 2.7 × 10^−12^; random effects: overall (weighted) ES = 0.21, 95% CI = 0.13–0.29, *z *= 5.3, *p *< 5.7 × 10^−8^]. Higher quality experiments produced a quantitatively larger effect size and a greater level of significance than lower quality studies. The number of contrary unpublished reports that would be necessary to reduce the level of significance to chance (*p *> 0.05) was conservatively calculated to be 87 reports. We explore alternative explanations and examine the potential linkage between this unexplained anticipatory activity and other results demonstrating meaningful pre-stimulus activity preceding behaviorally relevant events. We conclude that to further examine this currently unexplained anticipatory activity, multiple replications arising from different laboratories using the same methods are necessary. The cause of this anticipatory activity, which undoubtedly lies within the realm of natural physical processes (as opposed to supernatural or paranormal ones), remains to be determined.

## Introduction

Predicting the future is an essential function of the nervous system. If we see dark clouds and smell a certain scent in the air, we predict that rain is likely to fall. If we hear a dog bark, we predict that we will see a dog nearby. These everyday predictions are based on experience (e.g., memory) and perceptual cues. If even without experience and perceptual cues we could somehow prepare for important imminent events by activating the sympathetic nervous system prior to such events, this skill would of course be highly adaptive. More than forty experiments published over the past 32 years examine the claim that human physiology predicts future important or arousing events, even though we do not currently understand how such a thing could be accomplished. This meta-analysis examines a subset of these experiments allowing us to test the hypothesis that seemingly without experience and perceptual cues, human physiological measures anticipate what seem to be unpredictable future events by deviating from a baseline before an event occurs, in the same direction that they will continue to deviate after that event occurs. This is a controversial but important hypothesis. Thus, although there is no known mechanism for the effect reported in such studies, the implications of such an effect are far-reaching enough to justify a careful meta-analysis.

The studies we include in this meta-analysis make direct comparisons between pre-stimulus physiological activity measures using paradigms that produce a contrast in post-stimulus physiological activity between responses to stimuli from different categories. Two paradigms are used: (1) randomly ordered presentations of arousing vs. neutral stimuli, or (2) guessing tasks for which the stimulus is the feedback about the participant’s guess (correct vs. incorrect). In arousing vs. neutral stimulus paradigms, participants are shown, for example, a randomly intermixed series of violent and emotionally neutral photographs on each trial, and there is no *a priori* way to predict which type of stimulus will be viewed in the upcoming trial. In guessing tasks, on each trial participants are asked to predict randomly selected future stimuli (such as which of four cards will appear on the screen) and once they have made their prediction, they then view the target stimulus, which becomes feedback for the participant. Because participants perform at chance on these tasks, guessing tasks generally create a random distribution of events producing separable physiological responses that reflect brief states of positive arousal (following feedback indicating a correct guess) and negative and/or lower arousal (following feedback indicating an incorrect guess). Regardless of the paradigm, physiological measures [skin conductance, heart rate, blood volume, respiration, electroencephalographic (EEG) activity, pupil dilation, blink rate, and/or blood oxygenation level dependent (BOLD) responses] are recorded throughout the session, and stimulus times are usually marked in the physiological trace itself. These continuous data are later portioned according to a pre-determined “anticipatory period” designated for analysis (generally 0.5–10 s preceding stimulus presentation, depending on the temporal sensitivity of the physiological measure and the inter-trial interval). The portioned data are marked according to the type of stimuli they precede (arousing or neutral stimuli for the arousing vs. neutral paradigm, feedback indicating correct or incorrect guesses for the guessing paradigm). Pre-stimulus data are then compared across stimulus types.

It has been known for some time that arousing and neutral stimuli produce somewhat different *post-stimulus* physiological responses in humans (Lang et al., 1993, 1998; Cuthbert et al., 1996, 2000). However, what is remarkable is that many of the studies examined here make the claim that, for instance, the same physiological measure that yields a differential post-stimulus response to two stimulus classes also yields a differential pre-stimulus response to those same stimulus classes, *prior even to the random selection of the stimulus type by the computer*. Authors of these studies often refer to the effect as presentiment (sensing an event before it occurs) or unexplained anticipatory activity; we favor the latter terminology as it describes the phenomenon without implying that the effect truly reflects a reversal of the usual forward causality.

The primary value of this meta-analysis is that it tests a hypothesis that is different from those examined in most of the studies included in it. For the included studies, the hypotheses were, for the most part, bidirectional – namely, that the data would reveal a significant difference between physiological activity preceding two (or more) seemingly unpredictable stimulus types, regardless of the direction of that difference. A meta-analysis of these data would certainly be significant, as any deviation between the two physiological activity measures would produce a positive effect size (ES), in favor of a hypothesis that there is any difference between the measures. In contrast, adopting a more conservative approach, ours is a directional hypothesis: for paradigms producing post-stimulus effects differing between two or more stimulus types, and with randomized and theoretically unpredictable stimulus orders, the pre-stimulus difference between those same stimulus categories will have the same sign as the post-stimulus difference. In other words, we use meta-analytic techniques to test the hypothesis that the direction of pre-stimulus activity is predictive of the direction of post-stimulus activity, even when the stimulus category itself seems to be unpredictable^1^. To our knowledge, this is the first meta-analysis examining this phenomenon.

## Materials and Methods

### Inclusion and exclusion criteria

We took a relatively inclusive approach to ensure that all studies with negative and null results were included along with those supporting the hypothesis. A study was defined as a unique (not previously reported) examination of physiological responses to stimuli or events in one group of human participants; a report could include more than one study. Included studies were required to provide quantitative data or descriptive statistics reflecting physiological measures recorded during a period of time preceding stimulus presentation. This requirement excluded examinations of post-stimulus emotional responses that did not also report pre-stimulus activity. Further, only studies that marked stimulus event times using automatic (software) methods were included. Prospective (not *post hoc*) studies using human participants that fit these criteria and were reported in any language between 1978 and 2010^2^ were considered for inclusion if they provided dependent physiological variables during the anticipatory period from two or more classes of unpredictable stimuli (e.g., calm vs. arousing stimuli or feedback indicating correct vs. incorrect guesses) that produced different post-stimulus responses at the group level (e.g., a rise vs. no change in skin conductance), and they were published in English, German, Italian, or French (the languages spoken by the authors of this meta-analysis). The difference in the post-stimulus responses was usually obvious, but if the authors stated that there was no good post-stimulus separation of the physiological effect for a particular physiological variable and the data showed no clear quantitative post-stimulus difference between the two conditions being compared, we excluded those physiological variables as well. When post-stimulus responses were not reported, the authors were contacted to determine whether post-stimulus responses to the stimulus classes were different and if so, the direction and magnitude of the difference. If author contact was unsuccessful and no post-stimulus information was available, the study was not included, because we could not test our hypothesis without knowing the direction of the post-stimulus effect. Finally, the study could not report data that was exactly the same as those reported in another study by the same author (no duplicate studies were allowed; where duplicates existed, the first study reported in English was included). Any study passing these constraints and containing enough statistical information to calculate a *t*- or *z* score, or to directly calculate *d*_equivalent_ using the Rosenthal and Rubin formula (Rosenthal and Rubin, 2003), was included regardless of its level of peer review and the number or type of participants.

### Moderator analyses

Several potential moderators of apparent pre-stimulus differences were examined, including: study quality and whether an expectation bias analysis was performed. Because several authors have suggested that unexplained anticipatory activity is stronger among women than men (McCraty et al., 2004a; May et al., 2005; Radin and Lobach, 2007; Radin and Borges, 2009), we also examined the relationship between participant gender and effect size (ES). Finally, because a greater number of trials should result in a greater likelihood of participants implicitly learning any potential regularities in trial type, we examined the relationship between the number of trials performed by each participant and study ES. To assess quality, we calculated a combined quality score for each study based on level of peer review, type of random number generator (RNG), and whether the study reported results of an analysis of expectation bias. The combined quality scores ranged from 2.25 to 6.75. Studies with the highest quality scores were those that were published in peer reviewed journals, used hardware RNGs, and reported analyses of expectation bias (and found that expectation bias could not explain the effects). More details of the coding procedures can be found below (see Coding Procedures).

### Search strategies

All three authors were familiar with the unexplained anticipatory physiology literature, but to ensure consideration of studies about which we were not familiar, we performed broad web searches for studies reported between 1978 and 2010. We conducted the searches using PubMed, PsycInfo, Google Scholar, and the OAIster database from OCLC, which is useful for such gray literature searches. We also searched the archives of the *Journal of Parapsychology* for conference proceedings and published manuscripts, and contacted experts in the field (Dean Radin and Rollin McCraty) to request advice on finding additional studies. Our search terms were: presentiment + anomalous, anticipatory + physiology + anomalous, “expectation bias” + psi, “expectation bias” + presentiment, and “failure to replicate” + presentiment. Finally, all relevant referenced citations in each article we retrieved were retrieved as well and considered for inclusion. No manuscripts were excluded on the basis of titles or abstracts; all exclusions were made based on the most complete version of the manuscript available to our academic institutions. After discussing the studies located by each of the three authors, there were no disagreements as to the studies to be included and excluded.

### Coding procedures

The first two authors independently coded each of the studies before analyzing the results of the meta-analysis. The first author coded the studies before seeing the individual ES calculated for each study by the second author (see Statistical Methods). All ES disagreements were resolved by reviewing the calculation method for each study.

**Figure 1:**
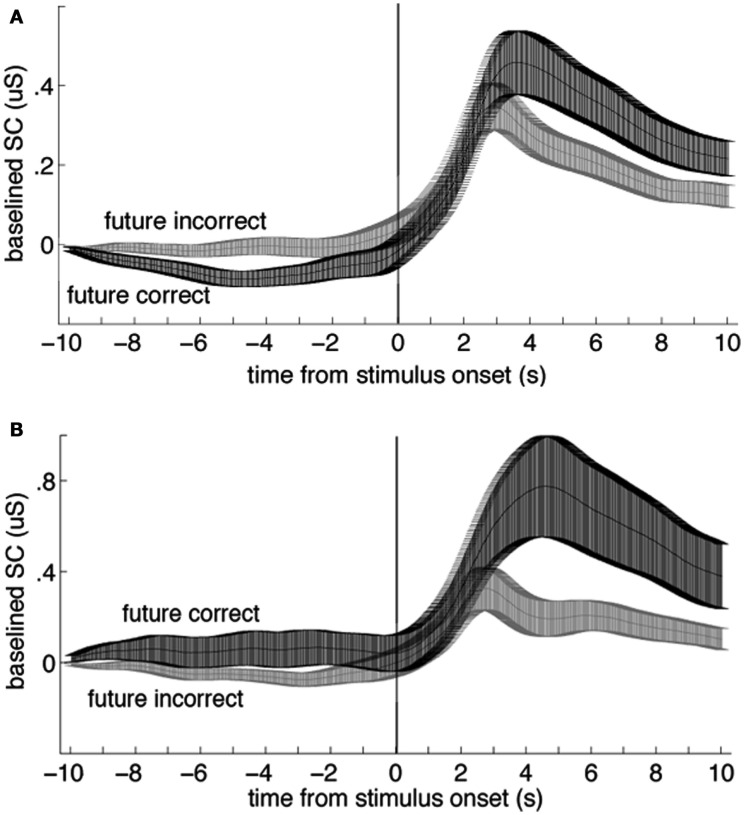
**Examples of data that would be coded with a negative (A) and a positive (B) sign for the effect size**. In each plot, the two lines represent group mean skin conductance baselined to the mean value from −11 to −10 s for trials in a four-choice guessing paradigm for which the upcoming event (vertical line at time zero) would be an indication of a correct vs. an incorrect guess (“future correct” or “future incorrect”). Participants performed at chance, thus there were about three times as many incorrect as correct responses. Across-participant standard error boundaries were calculated for each point and ±1 standard error of the mean (SEM) are marked with bars. **(A)** In the present analysis, these data would be coded with a negative sign for the effect size, because the pre- and post-event differences are in different directions (data from 54 females). **(B)** In the present analysis, these data would be coded with a positive sign for the effect size because the pre- and post-event differences are in the same direction (data from 30 males; note scale difference; data from Mossbridge et al., 2010). These data are not included in this meta-analysis because they arise from a *post hoc* analysis.

The first author coded the sign of the ES in all studies with a second pass (quality check) by the second author. The sign of the ES is one of the most critical parameters to be coded in any meta-analysis that tests a hypothesis that differs from some or all of the included studies. The sign of the ES could not be taken as the original sign given to the *t*- or *z* score reported by the authors, as often the authors were testing the bidirectional hypothesis that there was any significant difference (in any direction) between anticipatory physiological measures. In contrast, here we are testing a directional hypothesis that the pre-stimulus difference within a physiological measure will match the sign of the post-stimulus difference for that same measure. For this meta-analysis, the study ES was given a negative sign when the differences between the dependent variables did not match before and after the stimulus (e.g., Figure 1A), and a positive sign when they did (e.g., Figure 1B). We tested a unidirectional rather than a bidirectional hypothesis because a bidirectional hypothesis would almost certainly produce a significant overall result even if no individual results were significant, simply due to random variations in mean physiological measures.

We coded study quality weighted by three factors: peer review, use of hardware RNGs, and expectation bias analysis. Peer review is subjective and can be unreliable, and therefore is at best a guess about study quality (Casati et al., 2010). However, in our opinion the peer review process is more likely to catch errors in design and analysis than a publication process excluding peer review. Thus, we weighted study quality according to whether the report was peer reviewed and at what level. The type of RNG used to select stimuli was also considered in study quality scoring. This is because although pseudo-RNGs pass stringent tests of randomness, if they are not properly initialized, certain types of software (e.g., Matlab) will produce the same sequence of random numbers in each session, producing trial sequences that are consistent across participants. As a result, this could potentially create artifacts in the mean data produced by unintentional order effects that could appear to reflect unexplained anticipatory activity. Finally, one possible explanation for unexplained anticipatory activity is expectation bias, which arises when a random sequence including multiple repetitions of the same stimulus type (e.g., five non-arousing stimuli) produces an expectation in the participant that the next stimulus should be of another type (e.g., an arousing stimulus). Expectation bias can also arise when experimenters use non-equiprobable stimuli in an attempt to account for known emotional adaptation effects (e.g., a 2:1 ratio of calm to emotional images; McCraty et al., 2004a). Computational simulations and analytical efforts have shown that expectation bias can produce seemingly unexplained anticipatory activity (Dalkvist et al., 2002; Wackermann, 2002). Because of the potential explanatory power of expectation bias, analyses of expectation bias were performed in many of the studies we included in this meta-analysis. Analyses of expectation bias generally consist of determining whether the anticipatory effects on trials following contiguous trials of one stimulus type show a significant trend toward increasing with the number of preceding contiguous trials. We give a higher quality ranking to studies that report a negative result for an analysis of expectation bias, because their results are more likely to reflect an effect not based on experience or perceptual cues.

Initially, the first and second authors subjectively rated study quality, then chose a numeric ranking for each study. Intercoder agreement was fair, achieving a significant correlation between quality rankings from the first and second authors: *r* = 0.49, 95% CI = 0.12–0.82. Disagreements consisted of differences in perspective about level of peer review and level of expectation bias analysis, both factors that became contributors to the final quality score for each study. Arguments on both sides of these disagreements were reasonable, so we chose to calculate final quality scores as follows. The first two authors independently assigned numeric values to each study for three factors: level of peer review, with a higher level indicating what is likely to be a more thorough analysis and reporting process (1 = not peer reviewed, 2 = peer reviewed by referees for a conference, or meeting^3^, 3 = published in peer reviewed journal), expectation bias analysis, with a higher level indicating that the authors assessed whether expectation bias could explain the results [1 = no analysis or an analysis revealing that an expectation bias effect could account for the results (no study fit into this latter category), 2 = analysis described but data not shown, and any expectation effect could not account for the results, 3 = analysis described, data shown, and any expectation effect could not account for the results], and randomization, with a higher level indicating use of a RNG not subject to potential repeated sequences [1 = pseudo-random number generator (pseudo-RNG), 2 = hardware true RNG and pseudo-RNG, 3 = hardware true RNG alone]^4^. When a factor could not be established for a study, the study received the lowest score (1) for that factor. Values were combined using the following formula, with weights assigned to the factors: peer review + 0.75 × expectation analysis + 0.5 × randomization^5^. The weighting of the three factors was admittedly arbitrary, but this equation allowed us to weight the component scores in a way that reflects the primacy of peer review (which should catch troubles with expectation analysis and randomization), over expectation analysis (which should also catch randomization problems), over randomization. The resulting quality scores were averaged across the two coders to obtain the final quality scores reported here.

### Statistical methods

Each study reported physiological dependent variables for each of the stimulus categories the authors were comparing (Table A1 in Appendix); in our calculations of study ESs, we used the same dependent variables selected by each study’s authors. In no included study did we find evidence of data or subject exclusion, optional stopping, or data manipulation seemingly designed to produce an effect. No study reported statistics calculated using methods that were unfamiliar to us. However, several studies reported multiple statistical results from the same dataset, in these cases we took the conservative course of using the results that provided the smallest ES.

As is common in the analysis of psychophysiological data, in all included studies except one (Tressoldi et al., 2009), data from the pre-stimulus period were baseline-corrected to a time just preceding the start of the pre-stimulus period. Studies using electrodermal activity as the dependent measure either reported averaged baseline-corrected skin conductance preceding the stimulus in each category, counted the number of phasic electrodermal events preceding the stimulus or event in each category, calculated a proportion change score based on the change in skin conductance from a sample taken at the beginning of the pre-determined pre-stimulus period, or used random permutation software to attempt to sort the pre-stimulus electrodermal signals into categories. When the dependent measure was BOLD (e.g., fMRI studies), the authors calculated the mean BOLD signals during a pre-determined pre-stimulus or pre-event interval for each of the stimulus or event categories in a pre-determined region of interest (ROI). When heart rate was the dependent variable, studies reported either average heart rate during the pre-stimulus period, or a proportion change score based on heart rate change from the beginning of the pre-stimulus period. The studies using blood volume as the dependent measure reported a proportion change score from the beginning of the pre-stimulus period. The study using pupil dilation and blinks as the dependent measures presented pupil dilation change scores and proportion of data accounted for by blinks, respectively (Radin and Borges, 2009). Finally, the two included studies using event-related potentials (ERPs) calculated from EEG data either reported data from pooled electrodes (Fz, Cz, and Pz; Bierman and van Ditzhuyzen, 2006) or from the single electrode from which data were recorded (Oz; Radin and Lobach, 2007). When determining the post-stimulus direction of the effect for these studies, the average post-stimulus direction was considered rather than the direction of a particular component of the ERPs.

It is important to note that when determining the sign of the ES, the same measure(s) used to calculate the pre-stimulus effect was (were) used to determine the direction of the post-stimulus effect. In most cases, the direction was obvious from group data presented in figures or tables. In other cases, direction was determined as described above (see Inclusion and Exclusion Criteria).

We calculated a unique ES for each study, based on *t* or *z* scores reported or calculated from group averages of the pre-stimulus physiological activity measures chosen as dependent variables, for each of the stimulus categories. When comparing control and experimental conditions using independent (or uncorrelated) samples, the usual ES measure is $\text{ES}=\frac{{\bar{X}}_{\text{E}}-{\bar{X}}_{\text{C}}}{s_{\text{C}}},$ where E and C represent the experimental (here, more arousing post-stimulus response) and control (here, less arousing post-stimulus response) conditions, respectively, $\bar{X}$ is the sample mean, and *s*_C_ is the sample standard deviation for the control group. For paired data, two different formulas are commonly used to calculate ESs (see Appendix for details): (1) the ES used for the independent samples case corrected for the correlation between the dependent variables, $\text{ES}=t\sqrt{\frac{2\left(1-r\right)}{n}},$where *t* is the paired *t-*test statistic, *r* is the correlation between the values in the matched pairs and *n* is the number of pairs, or (2) the ES that measures the number of standard deviations the average difference between the variables falls from zero, where the standard deviation represents the variability of the differences. This latter measure is computed as $\text{E}{\text{S}}_{\text{D}}=\frac{t}{\sqrt{n}}\;\text{or}\;\frac{z}{\sqrt{n}},$ where *n* is the number of matched pairs. When differences are primary to a hypothesis and when there is no appreciable correlation between the dependent variables to be compared, the second method provides a smaller ES. When the correlation between the variables is larger than 0.5, the first method provides a smaller ES. To determine the appropriate method for use here, we calculated the correlations between dependent variables in the studies for which we had access to the raw data. We found that for baselined data, although the data were measured in pairs, the values were actually not correlated. However, for non-baselined data (Tressoldi et al., 2009), the correlation was very high (*r* = 0.95). Thus as a conservative measure, we used the first method to calculate the ES for Tressoldi et al., 2009, and the second method for all other studies. As a result, it was not necessary to calculate correlations for the remaining studies because the method of calculating ESs did not require it.

In terms of the original *t* and *z* scores from which ESs were calculated, different studies calculated statistics in disparate ways; for instance, several studies used bootstrap approaches to produce a *z* score, while others used a simple student’s *t*-test. In all cases in which *t* or *z* scores were reported, we used the score as reported by the authors and did not attempt to recalculate them, as the methods used by the authors were straightforward. For studies in which *t* or *z* scores were not reported, we calculated a *z* score based on the group averages for each measure in the study, then converted these averages to ESs using the equation shown above. For studies presenting single participant statistics, we averaged the *z* scores of each participant and calculated the ES of this mean *z* score as above. Statistics other than *t* and *z* scores (e.g., *F* or χ^2^ scores) were converted to ES (*d*; e.g., Borenstein et al., 2005). All of the reported analyses pertinent to the hypothesis of this meta-analysis were included in the calculation of ES for each study, and when more than one dependent variable was measured (e.g., heart rate and electrodermal activity), or when participants were split into more than one group (e.g., males and females), the ESs for each dependent variable were calculated, a sign was assigned to them, and then they were averaged. However, ESs for *post hoc* or for exploratory investigations of data already reported were not included in these calculations.

Standard error (SE) was calculated for each ES derived from baselined data with the formula $1∕\sqrt{n\text{,}}$ and 95% confidence intervals were calculated as *d* ± 2 × SE. Variability for the study not using baselined data (Tressoldi et al., 2009) was calculated using the formula $\text{SE = }\sqrt{\text{(}\frac{\text{1}}{n}\text{ + }\frac{d^{\text{2}}}{2n}\text{)2(1}-r\text{)}},$ (see Borenstein et al., 2009, p. 229). When calculating the overall statistics for the meta-analysis, ESs were weighted by the inverse of study variance to weight data from each participant approximately equally. This method gives a more precise estimate of the population effect than does weighting each study equally regardless of the number of participants (Borenstein et al., 2009).

In terms of models, the fixed-effect model is based on the assumption that the true ES is the same for all studies, while the random-effects model is based on the assumption that the true ESs differ across studies, and are sampled from a distribution comprising multiple different ESs. Both models are plausible here because we are not sure about the underlying distribution. Our heterogeneity analysis (see Results) reflects low heterogeneity across studies, suggesting that the fixed-effect model might be most appropriate. In the end, the models do not differ much; both give the same overall ES (see Results). To be complete, we report overall statistics for both models.

To test for “filedrawer effects” resulting from possible publication bias and/or selective reporting, we used two standard methods [classical fail-safe (Rosenthal, 1979) and Orwin’s fail-safe (Orwin, 1983] as well as a trim-and-fill analysis (Duval and Tweedie, 2000).

The statistical power of this meta-analysis is 0.90 assuming the true ES = 0.01 and variance = 0.002 (the observed variance in the random effects model; Hedges and Pigott, 2001). All meta-analytic statistical analyses (calculation of overall effect, tests of homogeneity, and trim-and-fill) were performed using Comprehensive Meta-Analysis version 2.2 (Borenstein et al., 2005). All other statistical analyses (correlations) were performed using the R statistical package version 2.11.1 (R-Development-Core-Team, 2011). All statistical tests were two-tailed, where relevant; no one-tailed tests were performed. Although ours is a unidirectional hypothesis, it is possible that the hypothesis is not only wrong, but the effect exists and is in the opposing direction. Thus two-tailed tests are justified. Results and statistical analysis were reported following Meta-Analysis Reporting Standards (American Psychological Association Publication and Communication Board Working Group on Journal Article Reporting Standards, 2008) and American Psychological Association (2010) statistical recommendations.

### Meta-analysis constrained to electrodermal data

One explanation for the anticipatory activity reported in the included studies is that researchers performed multiple analyses to find the dependent variable that produced the effect. This approach is more likely when the dependent variable arises from fMRI or EEG data, because multiple spatial and temporal locations can be used to define the dependent variables. However, electrodermal activity is a physiological endpoint that provides fewer opportunities for multiple analyses, because: (1) it offers only one spatial position (the point at which the electrodes were attached), and (2) the response time course of skin conductance measures is very sluggish (2–3 s), so that manipulating temporal parameters such as the duration of the pre-stimulus and baseline periods could influence the size of the result but it would only alter its direction if the two conditions produced phasic physiological responses that differed in phase during the pre-stimulus period. Most of the ESs of the studies included in this meta-analysis are based at least partially on electrodermal data (21 out of 26 studies, see Table A1 in Appendix). However, to reduce the likelihood that the results of this meta-analysis rely on multiple analyses, we performed a miniature meta-analysis of the subset of the included studies that included electrodermal activity as a dependent variable. For this meta-analysis, we re-calculated study ES based only on the electrodermal activity results (Table A1 in Appendix), where data were available, and performed the subsequent meta-analysis using the methods described above. The only study for which data were not available was Radin (1997), for which the author had combined several autonomic variables to create one *z* score. The author was contacted, but he no longer had access to the individual data from which the *z* score was drawn, so we excluded this study from the meta-analysis of electrodermal data.

## Results

### Overview of included studies

**Figure 2:**
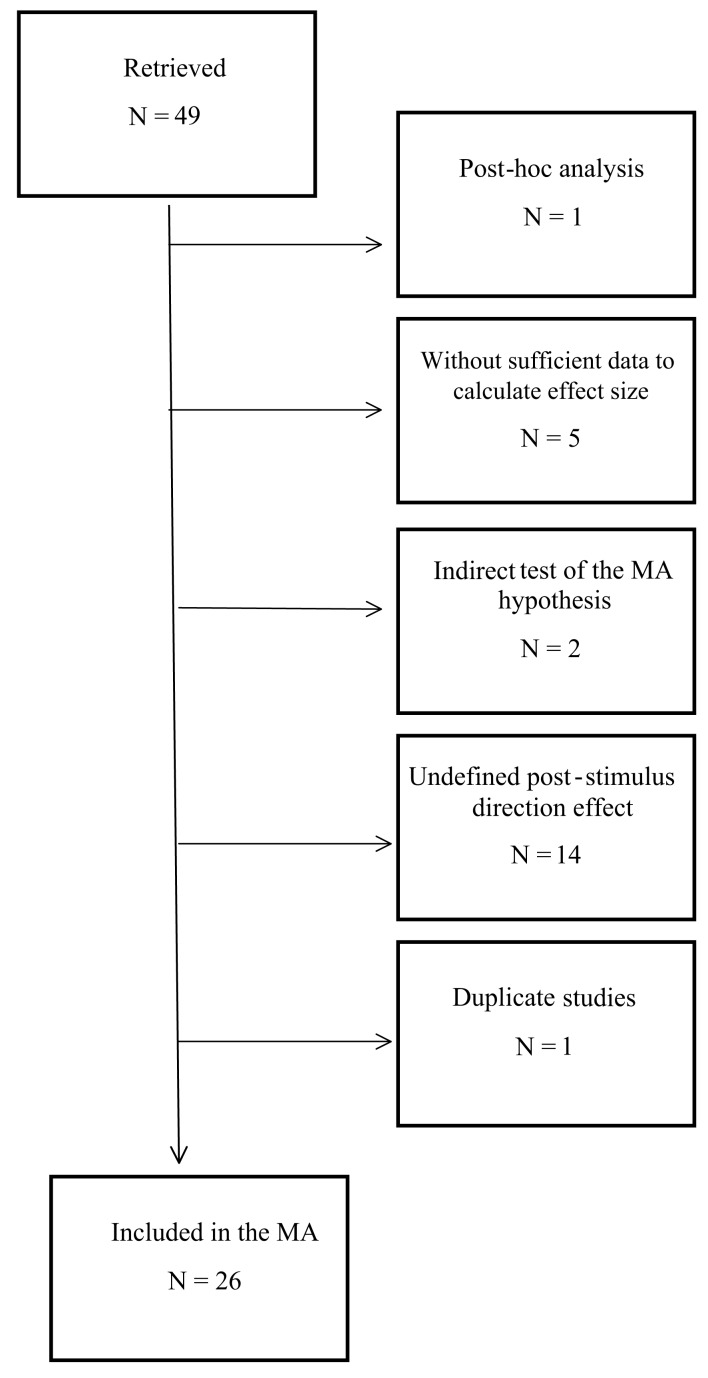
**Flowchart indicating the reasons for exclusion of 23 studies (also see Results, Overview of included studies)**.

Our search strategies retrieved 49 published and unpublished studies that initially seemed to fit our constraints (see Materials and Methods). However, 23 of these studies were excluded from the meta-analysis, for the following reasons (see Figure 2). Most of the analysis in one excluded study was *post hoc* (Vannini and DiCorpo, 2008), and another study was excluded because no group statistics were reported due to a null effect (Moulton and Kosslyn, 2008). The authors of this latter study were contacted for fMRI/BOLD statistics, but a whole-brain analysis was performed so no ROI statistics were available, and thus an ES could not be calculated. Four more studies from a single paper were excluded because no data were reported from which we could calculate study statistics; these studies were essentially descriptions of future studies that had not yet been performed (Bierman, 1997, studies 2–5). The remaining study in that paper (study 1; non-exploratory component) was included in the meta-analysis, but another study that reported exactly the same data was excluded to avoid duplication (Bierman and Radin, 1998, study 1). Two studies were excluded from another report because these studies used an indirect moderator-based categorization of participants that did not directly test the hypothesis of this meta-analysis (Tressoldi et al., 2009, studies 2 and 3). Several studies reporting significant or near-significant anticipatory effects were excluded because we could not determine the direction of the post-stimulus effect from either the reports themselves or from email correspondence with the authors (Bierman, 2007, fMRI component; Hartwell, 1978; Don et al., 1998; Lehman et al., 2000, 2001; McDonough et al., 2002; Parkhomtchouk et al., 2002; McCraty et al., 2004b; Sartori et al., 2004; Hinterberger et al., 2006, both studies; Tressoldi et al., 2005), or because the authors themselves stated that the direction of the post-stimulus effect was not clear and the data supported these statements (Mossbridge et al., 2010, study 2; Radin and Borges, 2009, study 2; see Materials and Methods). Note that this exclusion does not reflect a failing of these reports in any way. Most of these reports were designed to test a bidirectional hypothesis that did not depend on the direction of the post-stimulus effect and therefore post-stimulus effects were either not analyzed or not clearly reported. Finally, within the remaining included studies, several dependent variables reported within these studies were also excluded because they did not show appreciable post-stimulus effects (Bierman, 2000, study 3, animal vs. neutral comparison; Mossbridge et al., 2010 study 1, heart rate data; Mossbridge et al., 2010, study 3, heart rate data; see Materials and Methods) or the direction of the post-stimulus effect was not clear for that variable (Bierman and Scholte, 2002, female participants). Following these exclusions, 26 studies (Table A1 in Appendix) from seven different laboratories remained in our database. Note that most of the excluded studies showed significant or near-significant pre-stimulus differences between conditions. However, we could not use these differences to test our hypothesis without an appreciable post-stimulus difference between conditions with which to compare the directionality of the pre-stimulus difference.

### Overall effect size and statistical significance

The overall ES for all included studies is small, while the overall statistical significance is high [fixed effect: overall ES = 0.21, 95% CI = 0.15–0.27, *z *= 6.9, *p *< 2.7 × 10^−12^; random effects: overall (weighted) ES = 0.21, 95% CI = 0.13–0.29, *z *= 5.3, *p *< 5.7 × 10^−8^].

### Analysis of heterogeneity

Study ESs ranged from −0.138 to 0.67. Tests of homogeneity reflected relatively low heterogeneity (as defined in Huedo-Medina et al., 2006), *I*^2^ = 27.4, *Q* = 34.4, *p *> 0.098. This result suggests that most of the heterogeneity among ESs is due to sampling error, and there is little heterogeneity across studies.

### Investigation of potential moderators

**Figure 3:**
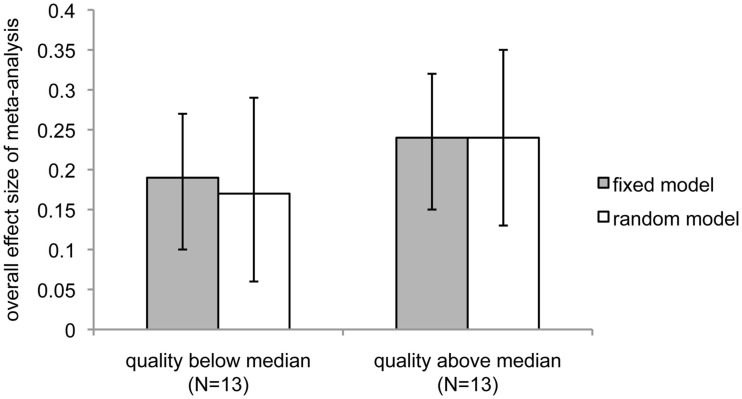
**Comparison of overall meta-analytic effect sizes between studies with quality scores above and below the median**. Dark bars show the overall effect size under the assumptions of the fixed-effect model; light bars indicate assumptions were those of the random-effects model. Error bars show 95% confidence intervals.

Although heterogeneity is low, we chose to examine several potential moderators of the effect. First, we examined study quality. Studies were scored for quality using a scoring procedure that encompassed level of peer review, type of RNG used, and whether or not an expectation bias analysis was performed (and if it was, whether expectation bias could have explained the results; see Materials and Methods). A median split was used to separate the studies into low (*N* = 13) and high (*N* = 13) quality experiments. If the overall statistical significance of the meta-analysis resulted from studies with low levels of peer review, pseudo- rather than true RNGs, and/or lack of examination of expectation bias, the above-median quality studies should have a non-significant ES. Instead, this analysis revealed that the higher-quality studies produced a higher overall ES and level of significance than the lower-quality studies (Figure 3; lower quality: fixed effect, overall ES = 0.19, 95% CI = 0.10–0.27, *z* = 4.3, *p *< 1 × 10^−5^; random effects, overall ES = 0.17, 95% CI = 0.06–0.29, *z* = 2.96, *p *< 0.002; higher quality: fixed effect, overall ES = 0.24, 95% CI = 0.15–0.32, *z* = 5.5, *p* < 2 × 10^−8^; random effects: overall ES = 0.24, 95% CI = 0.13–0.35, *z* = 4.4, *p *< 6 × 10^−6^). However, the correlation between quality score and ES was not significant (Pearson *r *= 0.21; 95% CI = −0.20–0.53), suggesting that studies with relatively poorer methodology and lower levels of peer review quantitatively, but not significantly, reduced rather than increased the overall ES of this meta-analysis.

**Figure 4:**
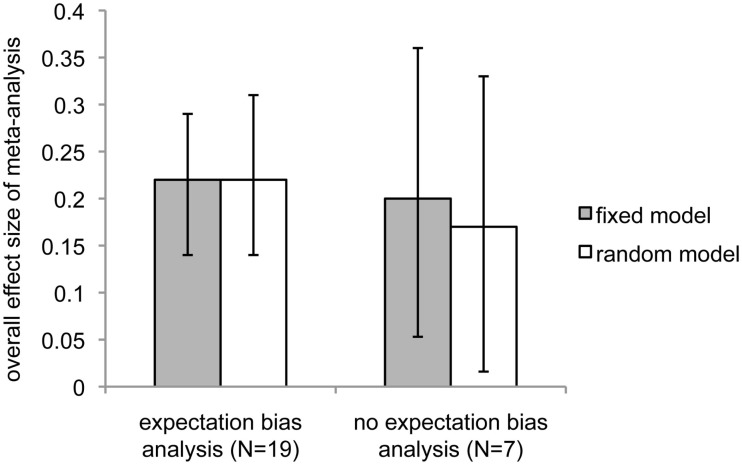
**Comparison of overall meta-analytic effect sizes between studies that performed expectation bias analyses and those that did not**. Dark bars indicate fixed-effect model; light bars indicate random-effects model. Error bars show 95% confidence intervals.

As pointed out previously (see Materials and Methods, *Guiding principles*), expectation bias could be a strong contender for a process that might explain these surprising results. Although none of the studies that included reports of expectation bias analyses were able to find evidence that expectation bias could explain the effects they reported, we chose to separately analyze expectation bias as a potential moderator of study ES. We separated the 26 studies into two groups: those that described performing expectation bias analyses (*N* = 19) and those that did not (*N* = 7). Reports of studies in which expectation bias analyses were performed produced a higher overall ES than reports that did not contain expectation bias analyses (Figure 4; expectation bias analysis: fixed effect, overall ES = 0.22, 95% CI = 0.14–0.29, *z* = 5.8, *p* < 4 × 10^−9^; random effects, ES = 0.22, 95% CI = 0.13–0.32, overall *z* = 4.7, *p *< 2 × 10^−6^; no expectation bias analysis: fixed effect, overall ES = 0.20, 95% CI = 0.09–0.31, *z* = 3.7, *p *< 0.0002; random effects: overall ES = 0.17, 95% CI = 0.016–0.33, *z* = 2.16, *p *< 0.016).

Finally, neither the ratio of male-to-female participants nor the number of trials in each study were related to the ES. The correlation between male-to-female participant ratio and study ES was not significant (Pearson *r *= 0.043, 95% CI = −0.51–0.51), and neither was the correlation between the number of trials performed by each participant in each study and study ES (Pearson *r* = −0.19, 95% CI = −0.53–0.17).

### Examination of potential reporting bias

Given such a surprising result, it is critical to investigate the potential influence of reporting bias. Skeptical mainstream scientific researchers would be unlikely to under-report negative results, as the effect examined here is controversial enough that reports of supporting evidence are not likely to further a mainstream scientific career. In contrast, there may be a sub-community of paranormal researchers who could be tempted to file away null or negative results. We think this unlikely for two reasons. First, many paranormal researchers were not investigating the directionally dependent hypothesis examined by this meta-analysis (see Materials and Methods), and would therefore be likely to publish results showing an effect opposing our hypothesis but consistent with their hypothesis. One example is a 2007 study (Bierman) in which the author reported anticipatory effects in multiple participant sub-groups and conditions that had a more moderate combined ES (∼0.26) when the directionality of each post-stimulus effect is not considered. The data reveal a small unexplained anticipatory effect such that physiological measures preceding calm stimuli differed significantly from those preceding emotional stimuli in some sub-groups and conditions. However, because for several of these measures during the pre-stimulus period the peak difference between these two measures was opposite in sign to the same difference taken during the post-stimulus period, here we coded the ES for those measures with a negative sign, resulting in an average ES for that study of −0.18. Thus, this study provided evidence against the hypothesis of our meta-analysis. The second reason we think publication bias is unlikely is that among paranormal researchers it is considered imperative to publish any null results. Once the Parapsychological Association Council adopted a policy that opposed only reporting significant results in 1975, null results began to be routinely reported (Bem and Honorton, 1994).

**Figure 5:**
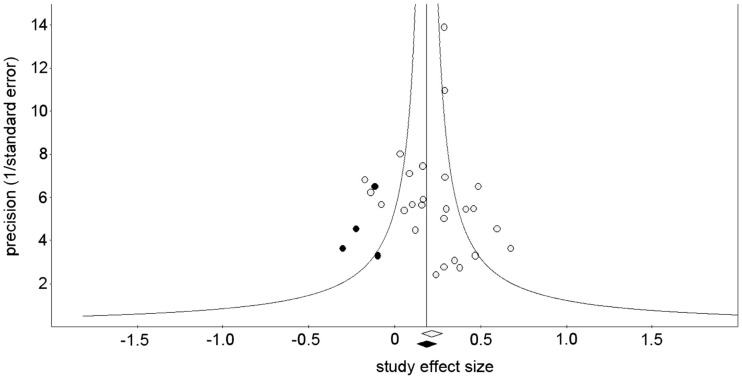
**Funnel plot showing the precision of the effect size estimate for each study (1/standard error) vs. the effect size of each study (open symbols), with four effect size estimates given by the trim-and-fill analysis (filled symbols)**. The open diamond at the base of the plot indicates the mean of the effect sizes before the trim-and-fill analysis was performed; the filled diamond indicates the mean of the effect sizes after the trim-and-fill analysis added the imputed studies.

On the other hand, it is still possible that the highly significant overall effect reported here could be explained by a “filedrawer effect” if negative findings were under-reported for some reason. To examine this possibility, we performed a trim-and-fill analysis (Duval and Tweedie, 2000) to estimate the overall ES if we had been able to include these presumably missing studies in the analysis. If this meta-analysis had included all the relevant studies, ESs would be distributed equally on either side of the mean overall effect. Thus, the trim-and-fill computation first eliminates any studies with higher ESs than the overall mean ES that are unbalanced with ESs lower than the mean (trim), calculates the new mean overall ES, then re-inserts the originally trimmed studies above the new mean ES and their arithmetic equivalents below the mean ES (fill). This method suggests that four studies with negative ESs are missing (Figure 5; Sterne et al., 2005). Under the fixed-effect model the trim-and-fill adjusted overall ES is 0.18 (95% CI = 0.12–0.24) and under the random effects model the trim-and-fill adjusted overall ES is 0.17 (95% CI = 0.09–0.23).

To further investigate the possibility that a persistent bias toward underreporting negative or null results could explain the significance of the overall effect, we used two methods to determine the number of contrary unpublished reports that would be necessary to reduce the level of significance to chance (*p *> 0.05). The classical fail-safe method (Rosenthal, 1979) provided a fail-safe number of reports of 265. A more conservative method (Orwin, 1983) gave a fail-safe number of 87 studies, assuming 0.05 as the “trivial” point estimate and 0.001 as the mean point estimate in missing studies.

### Meta-analysis constrained to electrodermal data

As discussed previously (see Materials and Methods), to reduce the likelihood that the observed effects could be related to researchers performing multiple analyses on physiological endpoints with many parameters, we performed an additional abbreviated meta-analysis of the included studies with ESs calculated only from results related to electrodermal activity (*N* = 20; for ESs, see SC ES column in Table A1 in Appendix). This analysis revealed a significant overall effect [fixed effect: overall ES = 0.17, 95% CI = 0.096–0.24, *z *= 4.5, *p *< 0.000004; random effects: overall (weighted) ES = 0.17, 95% CI = 0.074–0.27, *z *= 3.44, *p *< 0.0003, *I*^2^ = 36.8]. When the analysis was repeated on the subset of these ESs arising from studies that included expectation bias analyses (*N* = 14), the overall ES was quantitatively larger [fixed effect: overall ES = 0.19, 95% CI = 0.11–0.27, *z *= 4.6, *p *< 0.000003, *I*^2^ = 38.17; random effects: overall (weighted) ES = 0.19, 95% CI = 0.078–0.30, *z *= 3.3, *p *< 0.0005].

## Discussion

### Summary

The available data support the hypothesis tested by the current meta-analysis. Specifically, for paradigms producing post-stimulus physiological effects that differ among two or more intermixed and randomized stimulus classes, the group mean difference between physiological responses accompanying these stimulus classes seems to be in the same direction before and after stimulus presentation. For the 26 studies that fit our inclusion criteria (see Table A1 in Appendix), the estimated overall ES is small (most conservative estimate: 95% CI = 0.15–0.27), and is statistically significant. Though the ES is small, it is important to note that important scientific and health advances have been made by further examination of effects about half the size of this one (e.g., achievement scores vs. classroom size ES = 0.11, health outcomes vs. social support ES = 0.11; Rosenthal and Rosnow, 2008).

These results seem not to be an artifact of poor experimental design, as higher-quality experiments that addressed known methodological concerns (randomization and expectation bias analysis) produced a quantitatively if not significantly higher overall ES and level of significance than lower-quality studies. Further, the unexplained anticipatory effect examined here seems not to be due to expectation bias, as the overall effect was still highly significant when we included only those studies that reported expectation bias analyses and found that expectation bias could not explain the effects. Additional examination of other potential moderators of the effect revealed that the male-to-female ratio among study participants was not correlated with study ES; neither was the number of trials performed by each participant in a study correlated with ES.

Calculations to determine the number of contrary unpublished reports that would be necessary to reduce the level of significance to chance provided a fail-safe number of reports of 87 for the most conservative estimate. Seven laboratories contributed to the experiments included in this meta-analysis. Five more laboratories produced data that were related to our question, and many of them reported significant anticipatory effects, but they were excluded from this meta-analysis (see Materials and Methods). Together, this provides a rough estimate of the number of laboratories pursuing this type of work. Assuming all 12 laboratories have performed similar experiments but did not report them (a generous estimate) each of these 12 laboratories would have had to discard on average more than seven unpublished negative results to obviate the effect reported here. It is our opinion that this degree of selective reporting is unlikely to be found in all 12 laboratories, due to the time required to perform the pertinent experiments and the lack of funding available for them.

The results of the overall analysis are surprising, especially because in order to be inclusive we have combined data from multiple experimental paradigms and physiological measures that fit our constraints (see Materials and Methods). Almost certainly there are distinctions in responses between the arousing vs. calm stimulus paradigms and the guessing paradigms, and also between measures reflecting activity in different physiological sub-systems. Given this variability, it is remarkable that any effect is robust enough to be found across paradigms and physiological measures. However, future analyses are required to determine how task and measurement parameters influence this unexplained anticipatory activity.

In summary, the overall effect is small but statistically significant, seems not to be due to expectation bias, and is unlikely to be due to publication bias. Thus there seems to be a small, predictive anticipatory physiological shift in the seconds preceding apparently unpredictable stimuli. What could explain this effect?

### Possible explanations

One trivial explanation for the effect is that the compared stimulus categories did not differentially affect participants’ physiology, so it follows that the same random differences between physiological traces preceding the presentation of the two different stimulus classes also occur after stimulus presentation. However, our inclusion/exclusion criteria specifically required that all studies included in the meta-analysis use tasks presenting intermixed and randomized stimulus classes that produce appreciable physiological post-stimulus effects (see Materials and Methods).

A more reasonable explanation for the predictive anticipatory effect could be sensory cueing. Sensory cueing occurs when an experimenter allows information about a future stimulus to be obtained by the participant. Experiments using intentional sensory cueing were not considered for this meta-analysis, as the stimulus order would then not be random (see Materials and Methods). In all included studies, the experimenter was not aware of the order of the stimuli, as the next stimulus in each session was randomly selected on a trial-by-trial basis. However, unwitting sensory cueing might be a concern. One example could be the use of software that has many processing-intensive lines of code between the two lines in which the two stimulus classes are presented, which would produce a predictable differential delay between the presentations of stimuli from each class. Another example could be the unwitting presentation of sounds that are specific to each stimulus class, such as those made by the computer as it retrieves stimuli stored in two different hard drive partitions (Radin, 2004). Thus, sensory cueing is an obvious potential confound. Probably as a result of the awareness of this potential confound, every included study that reported information about the time of stimulus selection reported that stimuli were selected just before they were presented, not during the anticipatory period. Further, if there were some other subtle and distinct predictive cue associated with each of the upcoming types of stimuli, the cue would become more apparent with experience, which should result in a positive correlation between the number of trials performed by each participant and study ES, potentially indicating implicit learning of the stimulus order. Instead, there was a slight, non-significant negative correlation between the number of trials and ES. Taken together, these observations suggest that both sensory cueing and implicit learning are not good explanations for the anticipatory effect.

Another explanation includes the idea that the filtering of physiological data can produce artifacts, some of which can appear in the pre-stimulus period. A recent review of this phenomenon as demonstrated in EEG data showed that high-pass filters with low frequency cut-offs greater than 0.1 Hz can produce pre-stimulus effects that differ in direction from the post-stimulus response, assuming causal filtering is not used (Rousselet, 2012). For instance, a large positive post-stimulus response can appear to have a small negative pre-stimulus response. This meta-analysis includes two studies that used high-pass filters (Bierman and van Ditzhuyzen, 2006; Radin and Lobach, 2007). The authors of the first study reported a cut-off frequency of 0.01 Hz, and the authors of the second used a causal filter. However, the epoch duration in the first study was 3 s, a duration that could be long enough to produce a pre-stimulus artifact, even with the very low frequency cut-off of 0.01 Hz. In this case, the direction of this artifact would be in the opposite direction of the examined meta-analytic hypothesis, which is that the pre- and post-stimulus response differences are in the same direction. Further, the significance of the meta-analysis constrained to electrodermal data, which does not include any studies using high-pass filtering, suggests that filtering artifacts are not a good explanation for this seemingly anomalous anticipatory activity.

One might suspect that order effects could explain the predictive anticipatory effect described here. Order effects become more likely when fewer trials are performed, as order effects tied to a given stimulus order generally “wash out” when a greater number of randomly ordered trials are performed. Other order effects, specifically expectation bias, can occur when the two stimulus classes are not presented equiprobably, and a participant learns that one type of stimulus is more common among the potential stimuli. But we found that expectation bias could not explain the anticipatory effects in any of the studies in which these analyses were performed. However, different authors used different analyses, and it is critical to determine the most sensitive expectation bias analysis and to use that method in future studies of unexplained anticipatory activity. Other order effects, including forward priming, were not widely examined in these studies. Because experimenters randomized stimulus selection and order, because we assume that in most studies experimenters correctly initialized their RNGs and therefore presented a different stimulus order to each of their participants, and because most studies described tests of randomness passed by the RNGs, it is unlikely but not impossible that orders were consistent across most of the participant runs in one study. However, the chance of this occurring consistently in most of these 26 studies is vanishingly small, and even smaller in studies using hardware-number generators that do not require initialization. In spite of all these assurances, analyses of expectation bias and other order effects are critical to the clear understanding of the mechanisms underlying these predictive but seemingly anomalous anticipatory effects.

**Figure 6:**
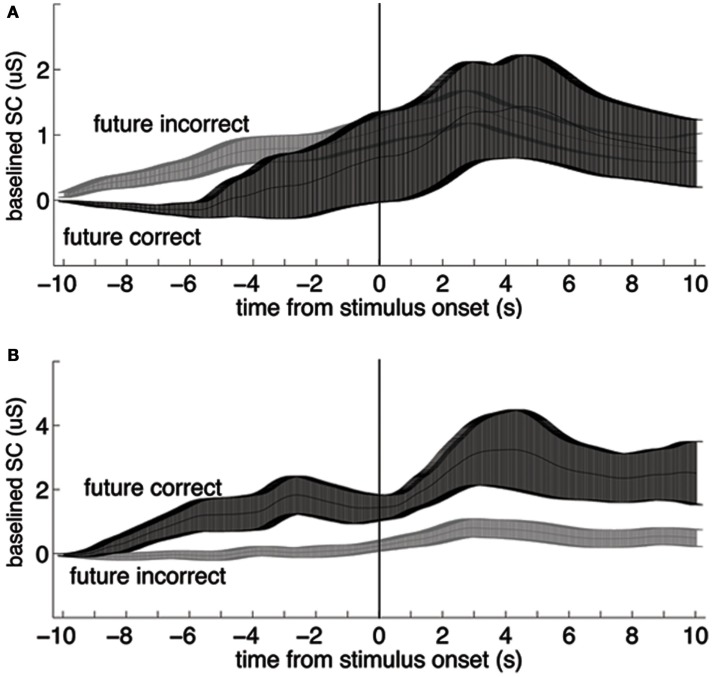
**Group mean traces of first trials only, from the same data set as in Figure 1**. Across-participant standard error boundaries were calculated for each point and ±1 standard error of the mean (SEM) are marked with bars. **(A)** Data from 54 females; **(B)** data from 30 males. The dependent variable was the average of skin conductance during the 10-s anticipatory period. Based on this conservative method, the comparison between skin conductance values on future correct and future incorrect guesses was not significant for females (*t*_52_ = −0.59, *p *> 0.554), however there was no appreciable post-stimulus difference between traces for the two types of trials, so the pre-stimulus difference is not a true test of our hypothesis. The same comparison was significant for males (*t*_28_ = 4.02, *p *< 0.0005, *d* = 1.49), who also showed a large and significant post-stimulus response (note difference in scales). The sex × correctness interaction was significant (*F*_80_ = 8.90, *p *< 0.004, ${\text{η}}_{p}^{2}=0.10$; data from Mossbridge et al., 2010; not included in this meta-analysis because they are from a *post hoc* analysis within that report).

One possible way to address order effects is to determine whether a between-participants anomalous anticipatory effect exists when participants perform only one trial in which a single randomly selected stimulus is presented. In such a paradigm, statistical power should be weaker due to the between-participant design, but the ES might be large enough to detect a significant anticipatory difference – unless these unexplained anticipatory effects are by-products of mundane order effects. Interestingly, a *post hoc* analysis performed on only first trials from several studies using the same guessing paradigm revealed that men produced a large significant anticipatory skin conductance effect (Figure 6; Mossbridge et al., 2010); this effect was not apparent in heart rate data from the same participants (data not shown), but there was also no differential post-stimulus effect for heart rate in these studies. Of course, replications of such single-trial studies are necessary, as is continued use of expectation bias analysis in future studies examining predictive anticipatory physiology in multiple-trial experiments.

One unfortunate possibility we must examine is either participant or experimenter fraud. Participant fraud can be easily ruled out – it is not clear how participants would be able to change their own physiology, even if they knew the direction in which they should change it in order to produce an effect. Although we did not find studies showing evidence of participant or data selection, optional stopping, or data manipulation, it is still possible that an unscrupulous experimenter in any discipline who is willing to commit what amounts to this sort of scientific fraud would be careful enough not to provide evidence of their fraud for the reader. Thus, no scientific venture can completely rule out fraud. Based on the strong significance of the overall ES estimated from the pertinent studies available between 1978 and 2010, to explain the predictive anticipatory effect examined here, such fraud would have to be widespread and undetected. We find the possibility of such massive collusion highly unlikely.

Another seemingly tractable explanation for the currently unexplained anticipatory effect is that some of the experimenters performing these experiments are using many methods of analysis and reporting the results for the one method that produces the biggest effect. This is an understandable approach in the early stages of the discovery of any phenomenon, as the work is necessarily exploratory because none of the factors influencing the effect are known. However, after performing an exploratory analysis, researchers would ideally settle on both a single paradigm and a single analysis method, then attempt to replicate their work using exactly the same paradigm and analysis. All of the authors of the studies we have examined here are presumably careful researchers. However, for any researcher, it is tempting to tweak paradigms when attempting a replication in order to obtain more information about the phenomenon than is provided by an exact replication. Unfortunately, this temptation may have produced a situation in which a single, replicable unexplained anticipatory physiology experiment with a well-defined paradigm and analysis method is not yet available. Such an experiment is critical for the future understanding of this currently unexplained effect. Because of the potential importance of the phenomenon, we encourage multiple researchers to pursue this aim in parallel.

Critically, this multiple-analyses hypothesis cannot fully explain the results of the present meta-analysis, as the hypothesis tested by most of the studies we examined was different from the hypothesis tested by this meta-analysis. Presumably, researchers would be biased toward methods that supported their hypothesis (any pre-stimulus difference) rather than methods that supported ours (a pre-stimulus difference matched in direction to the eventual post-stimulus difference). Thus, even if all researchers used analyses that maximized the likelihood of supporting their hypothesis (which we personally know not to be the case at least in our own work), and even if there were no real unexplained anticipatory effects, roughly half of the studies should have positive ESs and half should have negative ESs (relative to our hypothesis), which is clearly not the case. However, it is possible that unstated assumptions about the directionality of the effect could bias researchers toward finding analyses for which the post-stimulus effect matched the pre-stimulus effect. This sort of explanation could potentially explain the results. However, if this explanation is correct, it is unclear why the meta-analysis constrained only to electrodermal data produced a highly significant effect. As described previously (see Materials and Methods), the nature of electrodermal responses makes them less susceptible to multiple analyses as compared to fMRI and EEG responses, as only two parameters can be varied in an attempt to find an effect: pre-stimulus and baseline duration. These parameters are unlikely to influence the direction of the result, though they could influence its magnitude (see Materials and Methods). Despite these assurances, only repeated experiments with consistent paradigms and analyses will resolve the concern that multiple analyses could produce these unexplained anticipatory effects.

### Prevalence

The remarkably significant and homogeneous results of this meta-analysis suggest that the unexplained anticipatory effect is relatively consistent, if small in size. If so, the effect should be apparent in many experiments that present a series of emotional and calm stimuli. However, we agree with the scientists who design such experiments that both everyday experience and the second law of thermodynamics suggest a single direction for causality; causes normally precede effects. For these reasons, physiological effects preceding a subsequent cause are not generally assumed to exist, and are therefore not usually examined. In fact, one of the first analytical steps in most studies of physiological responses to distinct stimuli is to use the average of a time period preceding the stimulus as a baseline value. If this value is subtracted from all points in the physiological trace, such a baselining practice can effectively remove any evidence of a predictive anticipatory effect by zeroing out the anticipatory period (see below, Implications, for steps that can ameliorate this problem). Regardless of whether such a practice is followed, most researchers do not present much of the pre-stimulus period for comparison across conditions. For these reasons, predictive anticipatory effects may be both rampant yet invisible in mainstream psychophysiology results. Indeed, one study included in this meta-analysis that examined pre-stimulus data for three such experiments found anticipatory effects in all three mainstream studies investigated; one effect was significant (α = 0.05) and the other two were borderline (Bierman, 2000); all three went in the direction predicted by our hypothesis.

To determine whether other mainstream studies also contain evidence for similar anticipatory effects, we requested data from 14 researchers who published emotional physiology studies in non-parapsychology journals after 2000. Four offered to share data, but two of these four could not find the appropriate data files. Here we briefly report our analysis of the two data sets made available to us. For both data sets, multiple dependent variables were analyzed in the two published reports, which both focused on post-stimulus effects. Using the same methods we used to determine ESs for correlated data (see Materials and Methods), one study produced an overall ES of 0.021 (Ribeiro et al., 2007), and the other an overall ES of 0.343 (Lithari et al., 2010). Both ESs are in the same direction as our hypothesis, but we did not receive trial-by-trial data that would allow us to perform an expectation bias analysis. If not explained by expectation bias, results especially from the Lithari et al. (2010) study suggest that unexplained anticipatory activity may be under-reported in the physiology literature. Further, the results from the Lithari et al. (2010) study are independently statistically significant [*t*(27)], = 3.87, *p *< 0.0007), indicating that even when researchers are not looking for an unexplained anticipatory effect, such an effect can be found.

### Implications

As already briefly discussed, one possible explanation for the present results that may be made to fit the available data is that most researchers have an implicit assumption about the directionality of the effect and they used this assumption to select analysis methods that magnified the similarity between the pre- and post-stimulus effects as well as the ES. We consider this an unlikely but plausible explanation. Unlikely because we ourselves have analyzed our own data in multiple ways that produce larger pre-stimulus effects but feel constrained by scientific rigor to report only the results obtained with the originally selected analysis method. Further, we have had conversations with several of the other researchers whose studies we have examined here, and it is clear that their analysis methods were attempts at replications of previous analysis methods used by other researchers. However, the explanation is plausible because unexplained anticipatory activity is a phenomenon that is not well understood, and some researchers may feel justified in using multiple methods of analysis in order to better understand the effect. However, it is important to note that when researchers reported multiple statistical results from the same dataset we used the results leading to the smallest ES. Nevertheless, until this unexplained anticipatory effect is replicated multiple times using the same paradigm and method of analysis, we cannot completely rule out the multiple-analyses explanation. Further, there may be other explanations of which we are presently ignorant, but that will become clear over time. In the meantime, we speculate below about the implications of these results.

The most mundane implication of these results is that the existence of unexplained anticipatory effects could potentially either: (1) produce what seem to be null psychophysiological results due to baselining when in fact there is a significant pre-stimulus effect, or (2) produce significant psychophysiological results due to not baselining when there is a significant pre-stimulus effect accounting for the post-stimulus difference. Ideally, in future experiments the physiological variables preceding the stimuli or events of interest would be compared across stimulus classes first, before performing the usual baselining procedure. If there are significant baseline differences, then these differences should be reported in addition to any further post-stimulus effects observed after baselining.

More importantly, we feel that these predictive anticipatory effects constitute a fourth category in addition to three broad categories of anticipatory effects that have already been established in psychophysiology and neuroscience. The first category includes physiological anticipation of intentional motor activity, e.g., physiological anticipation of a willed movement begins at least 500 ms before the conscious report of the intention to move (Libet et al., 1983; Haggard and Eimer, 1999; Soon et al., 2008). The explanation for these effects is that human conscious experience is preceded by subconscious initiation of that experience (Libet et al., 1983). The second category consists of experiments for which the EEG signals during the pre-stimulus period from trials on which stimuli will later be detected differ significantly from the pre-stimulus signals from trials on which stimuli will later be undetected. The general explanation for these effects is that specific phases and/or amplitudes of neural oscillatory firing (Ergenoglu et al., 2004; Mathewson et al., 2009; Panzeri et al., 2010) facilitate detection (or non-detection) of an upcoming stimulus.

Recently, a third category of anticipatory effect, dubbed “preplay,” was discovered when the pre-maze activity of mouse hippocampal neurons was shown to mimic the activity recorded during and after being in the maze, even in mice for whom a maze was novel (Dragoi and Tonegawa, 2011). The authors also found that the firing patterns typically recorded in one maze are predictably different from those recorded in another maze. They offer the explanation that preplay patterns may reflect a sort of recycling phenomenon in which the hippocampus uses generalizable firing pattern templates from its recent history to code for an animal’s current spatial exploration experience.

For all three categories of anticipatory effects described above, the usual cause-preceding-effect assumption is sufficient to construct reasonable explanations for the observed phenomena. The seemingly anomalous anticipatory effects investigated in this meta-analysis could have some influence on the each of these three types of phenomena, but these unexplained anticipatory effects are not necessary to explain these three types of established anticipatory effects. Conversely, the three types of established predictive effects cannot explain the unexplained anticipatory activity examined here. Thus we suggest that unexplained predictive anticipatory effects belong in a category independent from, but potentially overlapping with, the three other categories of anticipatory effects already described.

In sum, the results of this meta-analysis indicate a clear effect, but we are not at all clear about what explains it. We conclude that if this seemingly anomalous anticipatory activity is real, it should be possible to replicate it in multiple independent laboratories using agreed-upon protocols, dependent variables, and analysis methods. Once this occurs, the problem can be approached with greater confidence and rigor. The cause of this anticipatory activity, which undoubtedly lies within the realm of natural physical processes (as opposed to supernatural or paranormal ones), remains to be determined.

## Conflict of Interest Statement

The authors declare that the research was conducted in the absence of any commercial or financial relationships that could be construed as a potential conflict of interest.

## Footnotes

^1^This hypothesis is likely too simplistic, in that some physiological measures may consistently reveal different pre-stimulus anticipatory directions depending on the valence of the upcoming stimulus (Rollin McCraty, personal communication) and/or participant characteristics such as gender. However, for a first meta-analysis of these phenomena, we thought it best to keep the hypothesis simple.

^2^We selected 1978 as the early cut-off because the first study we could find that was relevant to the meta-analysis was published in that year (Hartwell, 1978). We selected 2010 as the late cut-off because we performed this analysis in early 2011. We are aware of multiple related studies published in 2011 and 2012, all of which claim to show effects similar to those investigated by the studies we included in the meta-analysis, but because we did not perform an exhaustive search for studies published after 2010, we do not include these in the meta-analysis.

^3^While some peer review committees for conferences are very strict, it is our experience that some conferences have lax peer reviews or none at all. Therefore, to be conservative, we have given studies that appear in conference proceedings (and were not later published in a journal) a lesser score than those appearing in peer reviewed journals.

^4^It is important to note here that most authors using pseudo-RNGs reported tests of randomness passed by those generators. Thus, the sequences were clearly random. As a result, this ranking could be considered quite conservative, but we have chosen to take conservative steps wherever possible.

^5^It has been suggested that we use a fourth quality index reflecting the number of hypotheses tested by each study. However, for all studies included here, the main hypothesis was in regard to unexplained physiological anticipation of unpredictable future events. For studies testing other hypotheses, these hypotheses were either orthogonal to the main hypothesis (e.g., fMRI pattern differences in resting state activity for meditators and non-meditators), or were *post hoc* and therefore the data were not considered in this meta-analysis.

## Appendix

### Effect size measures for paired differences

When comparing control and experimental conditions using independent (or uncorrelated) samples, the usual effect size measure is $\text{ES}=\frac{{\bar{X}}_{\text{E}}-{\bar{X}}_{\text{C}}}{s_{\text{C}}},$ where E and C represent the experimental and control conditions, respectively, $\bar{X}$ is the sample mean, and *S*_C_ is the sample standard deviation for the control group. However, when the samples are paired, there are two different effect size measures that have been recommended, and which one makes more sense may depend on the situation.

#### Independent samples effect size

One possibility is to use the effect size used for the independent samples case, which can be estimated if the correlation is known or can be approximated: $\text{ES}=t\sqrt{\frac{2\left(1-r\right)}{n}},$ where *t* is the paired *t*-test statistic. We obtain this effect size measure used for independent samples from the paired *t*-test by noting the relationship between the standard deviation of the differences, σ_D_, and the standard deviation in the control group, σ_C_ which will be denoted by σ when we can assume that the experimental and control conditions have the same standard deviation:

$$\sigma_{\text{D}}^{2}=\text{Var}\left(X_{\text{E}}-X_{\text{C}}\right)=\sigma_{\text{E}}^{2}+\sigma_{\text{C}}^{2}-2r\sigma_{\text{E}}\sigma_{\text{C}}$$

where *r* is the correlation between the paired variables. When the experimental and control conditions have the same standard deviation, this becomes:

$$\sigma_{\text{D}}^{2}=\sigma^{2}+\sigma^{2}-2r\sigma \sigma =2\sigma^{2}\left(1-r\right)$$

Therefore, $t=\frac{\sqrt{n}\left(\bar{D}-0\right)}{s_{D}}=\frac{\sqrt{n}\left(\bar{D}-0\right)}{\sqrt{2s^{2}\left(1-r\right)}}=\sqrt{\frac{n}{2\left(1-r\right)}}\left(\frac{{\bar{X}}_{E}-{\bar{X}}_{C}}{s}\right)=\sqrt{\frac{n}{2\left(1-r\right)}}\text{ES}$

and

$$\text{ES}=t\sqrt{\frac{2\left(1-r\right)}{n}}$$

#### Effect size based on differences

The other possibility is to use $\text{E}{\text{S}}_{\text{D}}=\frac{\bar{D}-0}{s_{\text{D}}}$ where $\bar{D}$ and *s*_D_ are the mean and standard deviation of the differences. This effect size measures the number of standard deviations the average difference falls from 0, where the reference standard deviation represents the variability in the differences, rather than the variability in the individual groups. This effect size may make more sense if the differences are the primary measure of interest. The paired *t*-test statistic is $t=\frac{\sqrt{n}\left(\bar{D}-0\right)}{s_{\text{D}}},$ and note that $ES_{D}=\frac{t}{\sqrt{n}}$.

### Relationship between ES, ES_D_, and correlation

It is easy to see the relationship between these two effect sizes:

$$\text{ES}=t\sqrt{\frac{2\left(1-r\right)}{n}}=\frac{t}{\sqrt{n}}\sqrt{2\left(1-r\right)}=\text{E}{\text{S}}_{\text{D}}\sqrt{2\left(1-r\right)}$$

Therefore, the following relationships hold based on the correlation *r*:

When *r *> 0.5, ES* *< ES_D_

When *r* < 0.5, ES* *> ES_D_

In particular, when *r* = 0, $\text{ES}=\sqrt{2}\;\text{E}{\text{S}}_{\text{D}}$

**Table A1:** **Planned (not *post hoc*) studies of predictive anticipatory physiological signals from 1978 to 2010^a^**.

Citation	Study component	Stimuli	Psychophysiological measure(s)	Anticipatory period (s)	Effect size^b^	SE	Expect. bias analysis	Quality score	SC effect size^c^
Bierman (1997)	Study 1	Calm/violent images	Electrodermal activity	7.5	0.68	0.27	Yes	4.750	0.68
Bierman (2000)	Study 1	IAPS calm/emotional images	Electrodermal activity	7	0.17	0.0.17	No	3.250	0.17
Bierman (2000)	Study 2	IOWA gambling task	Electrodermal activity	not reported	0.47	0.30	No	3.250	0.47
Bierman (2000)	Study 3	calm/erotic images	Electrodermal activity	4	0.10	0.17	No	3.250	0.10
Bierman (2007)	Pilot study	Pleasant/unpleasant sounds	Electrodermal activity	3	-0.18	0.14	No	2.250	−0.18
Bierman and Radin (1998)	Study 2	Calm/violent images	Electrodermal activity	7.5	-0.08	0.17	Yes	4.250	−0.08
Bierman and Radin (1998)	Study 3	“	Electrodermal activity	7.5	0.16	0.17	Yes	4.250	0.16
Bierman and Scholte (2002)	Entire study	IAPS calm/violent/erotic images	BOLD in ROI: visual cortex	8.4	0.33	0.32	Yes	4.500	N/a
Bierman and van Ditzhuyzen (2006)	Entire study	Slot machine images (guessing task)	EEG/event-related potentials (ERP); entire pre-stimulus period pooled over Fz, Cz, and Pz	1	0.42	0.18	Yes	4.000	N/a
Broughton (2004)	Entire study	IAPS calm/emotional images	Electrodermal activity	3	0.03	0.12	Yes	5.500	0.033
Gillin et al. (2007)	Roulette	Roulette images (guessing task)	Electrodermal activity; heart rate	12	0.29	0.36	No	4.250	0.046
Gillin et al. (2007)	Business case	Business images (guessing task)	Electrodermal activity; heart rate	6	0.38	0.36	No	4.250	0.74
May et al. (2005)	Entire study	Alerting sounds	Electrodermal activity	3.5	0.29	0.14	Yes	6.750	0.29
McCraty et al. (2004a)	Entire study	IAPS calm/emotional images	Electrodermal activity; heart rate	6	0.29	0.20	Yes	4.375	0.13
Mossbridge et al. (2010)^d^	Exp 1	Calm IAPS images (guessing task)	Electrodermal activity	10	−0.14	0.16	Yes	4.750	−0.14
Mossbridge et al. (2010)^d^	Exp 3	“	Electrodermal activity	10	0.06	0.18	Yes	4.750	0.06
Radin (1997)	Entire study	Calm/emotional images	Electrodermal activity; blood volume	5	0.30	0.18	Yes	4.750	N/a
Radin (1998)	Entire study	IAPS calm/emotional images	Electrodermal activity; blood volume; heart rate	5	0.09	0.14	Yes	4.000	0.09
Radin (2004)	Study 1	Calm/emotional images	Electrodermal activity	5	0.60	0.22	Yes	5.000	0.60
Radin (2004)	Study 2	“	Electrodermal activity	5	0.16	0.13	Yes	5.750	0.16
Radin (2004)	Study 3	IAPS calm/emotional images	Electrodermal activity	5	0.49	0.15	Yes	6.000	0.49
Radin (2004)	Study 4	“	Electrodermal activity	5	0.24	0.41	Yes	5.000	0.24
Radin and Lobach (2007)	Entire study	Light flashes vs. no flashes	EEG/event-related potentials (ERP): Slow cortical potentials at Oz only	1	0.12	0.22	Yes	6.375	N/a
Radin and Borges (2009)	Exp 1	IAPS calm/emotional images	Pupil dilation	3	0.46	0.18	Yes	5.000	N/a
Spottiswoode and May (2003)	Entire study	Startle sounds vs. no sounds	Electrodermal activity	3	0.29	0.09	Yes	6.750	0.29
Tressoldi et al. (2009)	Study 1	Pleasant/alerting sounds	Heart rate	5	0.29	0.07	No	4.625	N/a

*^a^The entire database from which this table was drawn, including all moderator variables, is available upon request*.

*^b^As described in Section “Materials and Methods,” each ES was calculated separately for each dependent variable in a study, then averaged across all dependent variables to obtain one ES per study*.

*^c^These give, where possible, the effect sizes only for the electrodermal activity (skin conductance) dependent variables included in each study. These values were the values used for the meta-analysis constrained to electrodermal data*.

*^d^This report was submitted and peer reviewed, but was not included in the proceedings of the conference because the author could not attend the conference due to logistical problems. However, the same study was submitted and reviewed for the *2011 Annual Convention of The Parapsychological Association*. Although the 2011 conference referees did review the paper and it contained the same results as the 2010 paper, for quality scoring purposes, the two included studies from this report were scored as not peer reviewed*.
